# FURIN Stimulates NOTCH2 and NOTCH3 Pathways, Leading to Return of Function in Aged Cells

**DOI:** 10.3390/life16040588

**Published:** 2026-04-01

**Authors:** Peter L. Elkin, Jiaxing Liu, Jisaiah T. Wheeler, Thomas M. Suchyna, Wilma A. Hofmann

**Affiliations:** 1 Department of Biomedical Informatics, Jacobs School of Medicine and Biomedical Sciences, State University of New York at Buffalo, Buffalo, NY 14203, USA; jiaxingl@buffalo.edu; 2 Department of Physiology and Biophysics, Jacobs School of Medicine and Biomedical Sciences, State University of New York at Buffalo, Buffalo, NY 14203, USA; jisaiahw@buffalo.edu (J.T.W.); suchyna@buffalo.edu (T.M.S.); whofmann@buffalo.edu (W.A.H.)

**Keywords:** aging, FURIN, NOTCH signaling, myogenesis, skeletal muscle regeneration, GTEx

## Abstract

Background: Aging is accompanied by a progressive decline in skeletal muscle regeneration, largely due to impaired myogenic differentiation. The proprotein convertase FURIN is a key protease responsible for activating several signaling molecules, including precursors of NOTCH receptors, which regulate cell fate and differentiation. In this study, we investigated whether age-associated downregulation of FURIN contributes to impaired NOTCH2/3 signaling and myogenic function. Methods: An initial bioinformatics analysis of public scRNA-seq data from Genotype-Tissue Expression (GTEx) project indicated age-related expression of genes in the NOTCH signaling pathway. In vitro verification used early- and late-passage C2C12 myoblasts as a model of muscle cell aging to compare the expression of these genes. Late-passage C2C12 cells were transiently transfected with FURIN plasmid to assess restoration of differentiation potential, quantified by the fusion index, myogenic marker expression, and morphology. Results: Expression of FURIN, NOTCH2 and NOTCH3 was negatively correlated with age, whereas GZMB increased with age in GTEx dataset. Late-passage myoblasts exhibited impaired myotube formation, reflecting age-associated loss of myogenic capacity. Restoration of FURIN expression in aged myoblasts was associated with reduced GZMB levels, increased expression of embryonic myosin heavy chain IGF1, and partial recovery of myogenic differentiation and myotube formation. Conclusions: These findings suggest that age-associated loss of FURIN contributes to impaired NOTCH2/3 pathways and myogenic dysfunction. Overexpression of FURIN partially rescues the myogenic phenotype and increases expression of early myogenic markers in aged cells, identifying FURIN as a potential regulator of muscle regenerative capacity during aging. We suggest FURIN as a promising candidate target for further investigation into the mechanisms driving aging or age-related decline.

## 1. Introduction

Aging is a time-related process causing the deterioration of functions in tissues and organs. Cellular senescence is a state of cell cycle arrest where cells lose the capacity to divide while maintaining metabolic activity. The accumulation of senescent cells is closely related to aging and age-related dysfunction of tissues and organs [1].

The NOTCH signaling pathway is an evolutionally conserved pathway that is involved in the regulation of the development processes of the human body. The NOTCH signaling cascade is triggered when a ligand expressed on another cell binds to the NOTCH receptor. After binding, the NOTCH receptor is cleaved by ADAM, and its intracellular domain is released and trafficked into the nucleus, where it forms a transcription complex with other proteins and regulates the expression of downstream genes [2]. Previous studies have shown an emerging role of the NOTCH pathway in the regulation of aging and that the dysregulation of NOTCH signaling has been implicated in several human development disorders and many cancers [3]. In muscle aging, NOTCH is indispensable for muscle satellite cell maintenance and regeneration. For example, the diminished activation of NOTCH may contribute to cellular senescence in skeletal muscle stem cells, and the myogenic differentiation capacity of satellite cells was achieved via forced activation of NOTCH [4,5].

Furin is a proprotein convertase (PC) that proteolytically cleaves precursor molecules into their mature forms [6]. In the NOTCH signaling pathway, the nascent precursors of four members of the NOTCH family (Pre-NOTCH1, Pre-NOTCH2, Pre-NOTCH3, and Pre-NOTCH4) undergo posttranslational modifications in the endoplasmic reticulum and Golgi apparatus. FURIN is responsible for the maturation of NOTCH by catalyzing the proteolytic cleavage of Pre-NOTCH in Golgi [7,8,9]. FURIN knockdown was also found to decrease the expression of NOTCH-related proteins [10]. Therefore, FURIN plays a curial role in the activation of NOTCH signaling.

Understanding the molecular mechanisms that underlie aging is a longstanding goal in biomedical research, with the aim of mitigating age-associated disorders and functional decline. The purpose of this study is to identify innovative biomarkers within the NOTCH pathway for aging and age-related diseases, as well as potential therapeutic targets. In this study, we initially identified gene candidates with chronologically age-related expression patterns in silico and propose a hypothesis of Furin as a therapeutic target based on correlation. A subsequent in vitro experiment on a C2C12 aged cell model demonstrates that restoration of FURIN expression is sufficient to partially reverse age-associated myogenic dysfunction in an in vitro model. Sarcopenia, age-related muscle loss, is a major public health concern in aging populations, contributing to frailty, reduced independence, and increased risk of adverse outcomes. Sarcopenia is influenced by multiple factors, including aging, physical inactivity, poor nutrition, and chronic disease. Its growing prevalence underscores the need for early identification and intervention to preserve functional capacity and quality of life in older adults [11,12]. A central contributor to sarcopenia is the decline in satellite cells, the resident muscle stem cells responsible for repairing and regenerating myofibers. With aging, satellite cells decrease in number and exhibit impaired activation, proliferation, and differentiation through a number of intrinsic and extrinsic factors [13,14]. Here we present molecular and functional evidence that Furin has the potential to restore the myogenic potential of aged myoblasts.

Many disorders are more prevalent or more severe with advancing age, reflecting shared age-associated changes across tissues. In addition to skeletal muscle, prior studies have reported that reduced FURIN activity during aging is associated with altered processing of proteins, such as the amyloid precursor protein and α-synuclein, which are linked to neurodegenerative disorders, including Alzheimer’s and Parkinson’s disease [15,16,17,18,19]. Together, these findings motivate further investigation into age-associated regulation of FURIN across multiple age-related disease contexts.

## 2. Methods

### 2.1. Bioinformatics Analysis

An initial retrospective analysis was conducted on the Adult Genotype-Tissue Expression (GTEx) Portal dataset, which contains scRNA sequencing data from 701 human individuals aged 20–79. Samples were divided into 6 age groups with a time granularity of 10 years (e.g., 20–29, 30–39, 40–49, 50–59, 60–69, 70–79). The RNA read count data of two skin tissues of the lower leg (sun-exposed) and the suprapubic region (not sun-exposed) were used for differential expression (DE) analysis, as skin’s high turnover can amplify aging signature. Median ratio normalization and DE analysis were conducted in R with the DESeq2 package [20]. DE analysis across all age groups was performed on NOTCH-related mRNA using the likelihood ratio test. We report the Benjamini–Hochberg FDR-adjusted *p*-values, and genes with FDR < 0.05 were considered significant. The list of NOTCH-related mRNA used was retrieved from Reactone [21,22,23]. For the significant DE genes identified, normalized expression counts were extracted from the DESeq2 object. Subsequent correlation tests were performed using the ‘stats’ package between gene read counts and age groups to identify biomarkers with chronologically age-related expression patterns, where absolute rho-values of 0.1 and two-side *p*-values of 0.05 were used as thresholds. Analysis of genes related to pre-NOTCH was also conducted. The list of molecules investigated can be found in Table S1. The analysis was performed with R. All codes are available via the Github repository.

### 2.2. Cell Culture

Murine skeletal myoblasts (C2C12) [24] were kindly provided by Dr. Thomas M. Suchyna (Department of Physiology and Biophysics, University at Buffalo, Buffalo, NY, USA). The cells were maintained in growth medium (GM) consisting of high-glucose Dulbecco’s Modified Eagle Medium (DMEM, Gibco, Grand Island, NY, USA) supplemented with 10% (*v*/*v*) fetal bovine serum (FBS, Gibco, Grand Island, NY) and 1% (*v*/*v*) penicillin/streptomycin (Gibco, Grand Island, NY, USA), at 37 °C with 5% CO_2_. Cells were maintained at a confluency of less than 80% and subcultured every other day using 0.25% trypsin ethylenediaminetetraacetic acid (EDTA) solution suitable for cell culture (Sigma-Aldrich, St. Louis, MO, USA). Late-passage (LP) cells, older cells at passage 55–60, have largely lost the ability to differentiate into myotubes, as described before [25], while early-passage (EP) cells, younger cells, were used between passages 10 and 15. For cell counting, cells were trypsinized, stained with 0.4% trypan blue (Thermo Fisher, Waltham, MA, USA), and counted using an automatic cell counter (TC20, Bio-Rad, Hercules, CA, USA).

### 2.3. Gelatin Coating

For transfection and differentiation experiments, cells were seeded in gelatin-coated 6-well plates or on gelatin-coated glass coverslips in 12-well plates. For gelatine coating, gelatin type A from porcine skin (Sigma-Aldrich, St. Louis, MO, USA) was dissolved in water at 0.2% (*w*/*v*), sterilized by autoclaving, and added to the respective tissue culture dishes at 0.2 mg/cm^2^ for 1 h at 37 °C. The solution was then aspirated, and the gelatin-coated dishes were dried in the laminar flow hood before seeding cells.

### 2.4. Differentiation Experiments

To initiate myoblast differentiation into multinucleated myotubes, myoblasts were seeded on gelatin-coated 6-well plates or gelatin-coated glass coverslips at a density of 30,000 cells/cm^2^ and allowed to reach ~90% confluency. Differentiation was induced by washing the cells twice in PBS before adding differentiation medium (DM) consisting of high-glucose Dulbecco’s Modified Eagle Medium (DMEM, Gibco, Grand Island, NY, USA) supplemented with 2% (*v*/*v*) horse serum, 10 µg/mL insulin (Gibco, Grand Island, NY, USA) and 1% penicillin/streptomycin (Gibco, Grand Island, NY, USA) for up to 6 days.

Fusion index (FI) of myotubes (MT) was calculated by dividing the number of nuclei in the myotubes by the total nuclei in a field of view ($FI=\frac{\mathrm{number nuclei in myotubes}}{\mathrm{total number of nuclei}}*100\%$). The FI values are reported as the mean ± standard deviation (std) of at least three fields of view (>700 total nuclei).

### 2.5. Plasmids and Transfection

The mus musculus 6XHis-Furin construct pcDNA3.1-mPCSK3 was a gift from Liming Pei (Addgene plasmid #122674; http://n2t.net/addgene:122674 (accessed on 20 February 2026); RRID: Addgene_122674). Control transfections were performed using the pCMV-myc vector (Invitrogen, Carlsbad, CA, USA). At 12 h after plating on gelatin-coated surfaces, cells were transfected with the DNA constructs using Lipofectamine 3000 (Invitrogen, Carlsbad, CA, USA) by adding the DNA–Lipofectamine solution directly to the GM, without changing the culture medium to serum-free Opti-MEM (Gibco, Grand Island, NY, USA) to avoid induction of myotube formation. At 48 h after transfection, differentiation was induced as described above. At the indicated time points, cells were processed for immunofluorescence imaging or for quantitative Real-Time PCR (qRT-PCR).

### 2.6. Quantitative Real-Time PCR (qRT-PCR)

Total RNA was isolated from cells using Trizol^®^ reagent (Invitrogen, Carlsbad, CA, USA) following the manufacturer’s instructions. First, 1 µg total RNA from each tissue sample was reverse transcribed into cDNA using the iScript cDNA Synthesis Kit (Bio-Rad, Hercules, CA, USA) according to the manufacturer’s instructions (Invitrogen, Carlsbad, CA, USA). qRT-PCR was performed using the iQ SYBR Green Supermix and the iCycler iQ Real-Time PCR Detection System (Bio-Rad, Hercules, CA, USA). Table 1 shows the primers used in qRT-PCR. Primers were purchased from Integrated DNA Technologies (IDT; Coralville, IA, USA).

Relative mRNA expression was calculated by the ΔCT method. Triplicates were normalized to the average of the GAPDH housekeeping gene under the same conditions or to endogenous expression, as detailed in the figure legends. The values are shown as the mean ± std of triplicates in each experiment.

### 2.7. Immunofluorescence Staining and Microscopy

Cells grown on glass coverslips were fixed with 4% paraformaldehyde for 15 min, permeabilized with 0.1% Triton X-100 for 7 min, and then blocked with 5% BSA for 1 h. Incubations with the primary antibodies (α-myoin II heavy chain; MYH4 [MyHC-IIb] monoclonal antibody [MF20]; Invitrogen, Carlsbad, CA, USA) were conducted at 4 °C overnight, followed by incubation for 1 h at room temperature with the appropriate secondary antibodies conjugated to FITC (Jackson ImmunoResearch Laboratories, West Grove, PA, USA). Rhodamine-conjugated phalloidin was used for F-actin staining (Cytoskeleton Inc., Denver, CO, USA). Coverslips were mounted with Prolong antifade containing 4′,6′-diamino-2-phenylindole (DAPI; Invitrogen, Carlsbad, CA, USA). Images were taken on a Leica DM 6B microscope (Deer Park, IL, USA) and processed using Leica (Leica Application Suite X) and ImageJ 1.54p software (National Institutes of Health, Bethesda, MD, USA)

### 2.8. Total Cell Extract Preparation and Immunoblotting

Total cell extract was prepared as described [26]. For detection, proteins in total cell extract from an equal number of cells or 30 µg per sample were separated by SDS-PAGE and transferred onto nitrocellulose membrane. After the transfer, the blots were either probed intact or cut according to the size of the respective probed protein and probed with specific antibodies. The immunoreactive bands were detected by enhanced chemiluminescence.

Antibodies: α-β-actin (MilliporeSigma, Burlington, MA, USA); α-Furin (clone 1P6N8; MilliporeSigma, Burlington, MA, USA); α-MyHC_emb (MYH3, Abcam, Waltham, MA, USA); MyHC_IIa (MYH2; MilliporeSigma, Burlington, MA, USA). Peroxidase-conjugated secondary anti-mouse or anti-rabbit antibodies were from Jackson ImmunoResearch Laboratories (West Grove, PA, USA).

## 3. Results

### 3.1. Bioinformatics Analysis Discovered Age-Related Expression Alternation of NOTCH-Related Gene

We used the GTEx database and compared people in their 20s to those in their 30s, 40s, 50s, 60s and 70s (701 individuals). Our initial in silico research focused exclusively on NOTCH pathways given the well-established knowledge of the importance of NOTCH in aging [2,3,4,5]. Differential expression (DE) analysis was performed to identify genes with significant expression changes among the age groups in both tissues. In order to further understand the effect of chronologic age, we conducted Spearman correlation test on each significantly differentially expressed gene. The two-step tests revealed that the expression levels of NOTCH receptor genes *NOTCH2* and *NOTCH3* were negatively associated with age in both tissues; in contrast, downstream gene *GZMB* was positively associated with age in both tissues.

**Figure 1:**
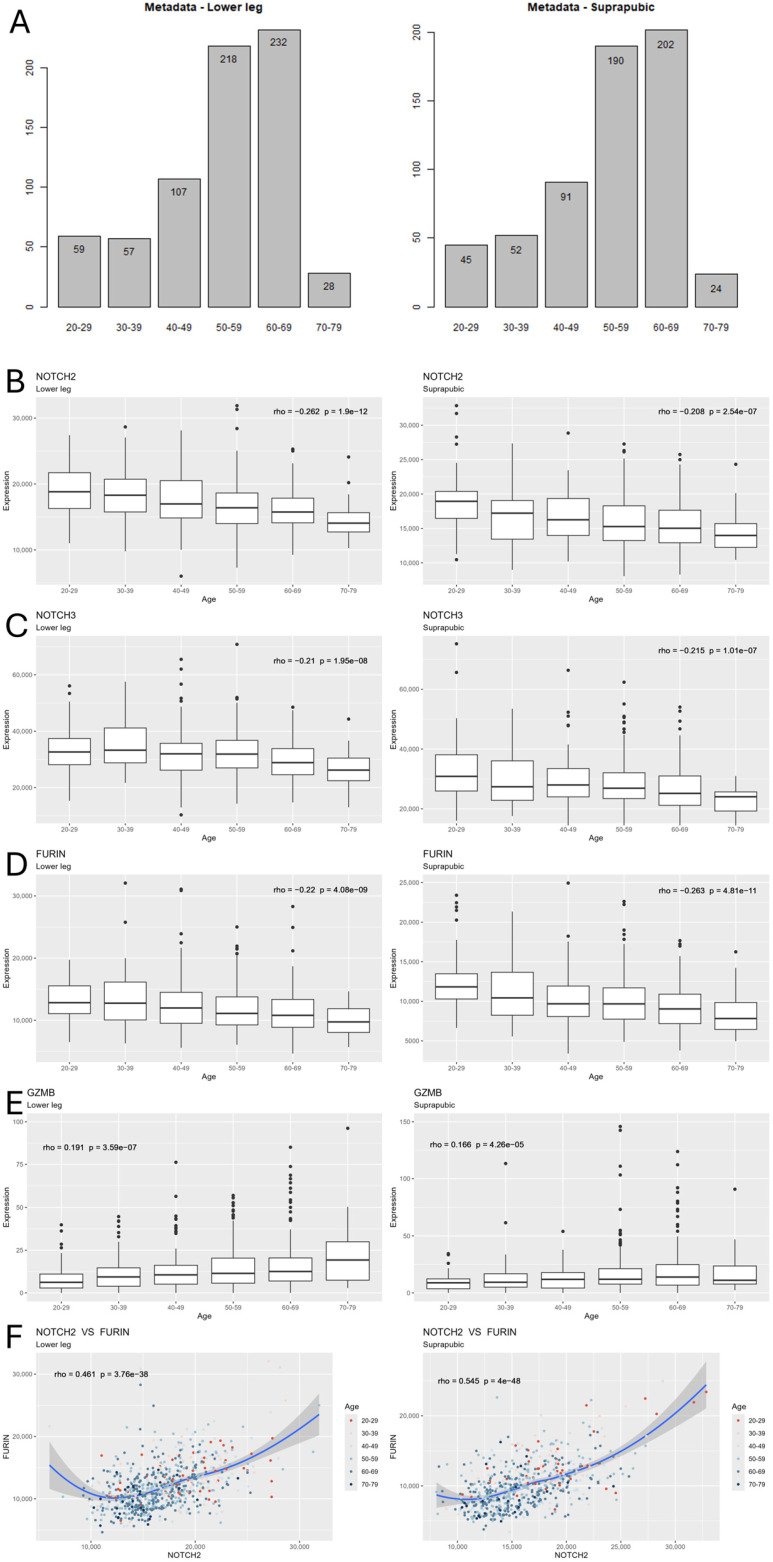
Bioinformatic analysis revealed age-related downregulation of FURIN, NOTCH2 and NOTCH3. (**A**) Age distribution of lower leg (**left**) and suprapubic region (**right**) samples. (**B**–**E**) Gene read counts of *NOTCH2*, *NOTCH3* and *FURIN* were negatively associated with age, whereas *GZMB* was positively associated with age in both tissues. Statistical analysis was performed using Spearman. (**F**) Expression of *NOTCH* was positively associated with *FURIN*. Statistical analysis was performed using Pearson. Rho and *p*-values are shown in the figures.

To explore potential upstream mechanisms that may contribute to the observed dysregulation and identify potential targets for NOTCH restoration, we subsequently investigated genes related to the pre-NOTCH pathway, a regulatory pathway that governs the maturation of NOTCH receptors. DE analysis and correlation tests revealed that the convertase gene *FURIN* was expressed in a similar age-related pattern as *NOTCH2* and *NOTCH3*, where it was negatively associated with age. Additionally, the Pearson correlation test demonstrated a strong positive correlation between *FURIN* and *NOTCH2* and *NOTCH3* in both tissues (see Figure 1).

Based on the correlation between *FURIN* and *NOTCH2/3*, their age-related downregulation, and the biological mechanism that Furin protease is involved in the cleavage of NOTCH precursors, we hypothesized that age-related downregulation of FURIN can lead to a decrease in NOTCH receptors, leading to coordinated alterations in NOTCH signaling and downstream buildup of age-associated gene products, such as GZMB.

### 3.2. Expression of FURIN, NOTCH2 and NOTCH3 Is Negatively Associated with Aging, and Expression of GZMB Is Positively Associated with Aging in the C2C12 Murine Myoblast Model

To determine the functional relevance of the observed association of these genes with age, we used the established C2C12 in vitro model of age-related muscle loss. Sarcopenia, a progressive decline in muscle mass and function that is most commonly but not exclusively associated with aging, is linked to decreased mobility, increased risk of obesity, decrease in metabolic function and the development of associated comorbidities and mortality [12,27,28].

An established in vitro model to analyze age-related muscle loss is the use of the late-passage C2C12 murine myoblast cell line [24]. Upon serum removal, early-passage C2C12 myoblasts differentiate into functional multinucleated myotubes [29]. However, serial passaging of C2C12 cells leads to a gradual loss of myogenic differentiation similar to aging muscle cells, and three-dimensional bioengineered skeletal muscle constructs of late-passage C2C12 demonstrated a replication of the phenotype of aged skeletal muscles, demonstrating the suitability of this model system to study sarcopenia-related pathways in vitro [25,30,31].

**Figure 2:**
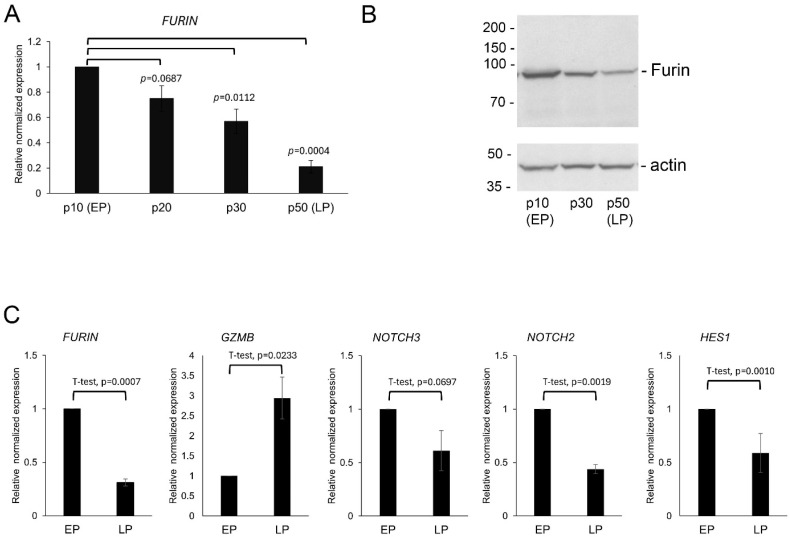
Expression of *FURIN*, *GZMB*, *NOTCH2*, *NOTCH3* and *HES1* was altered in late-passage myoblasts. Quantitative real-time PCR analysis of mRNA expression levels of the indicated genes normalized to *GAPDH*. (**A**) Progressive decrease in *FURIN* mRNA expression during serial passaging of C2C12 cells. (**B**) Representative immunoblot of total cell extract from C2C12 myoblasts at the indicated passage number showing a progressive decrease in Furin protein expression (**C**) *FURIN*, *GZMB*, *NOTCH2*, *NOTCH3* and *HES1* expression in early- (EP) and late- (LP) passage C2C12 myoblasts. EP expression levels were set to 1. Results are presented as means *±* standard deviation; *n* = 3, *p*-values in expression level changes in LP as compared to EP are indicated.

**Figure 3:**
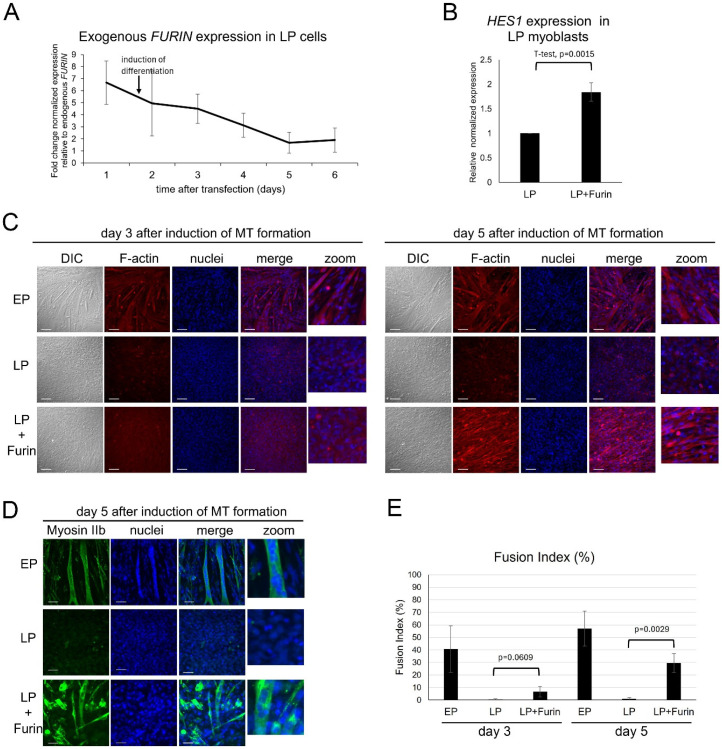
Expression of exogenous Furin-restored myotube formation in late-passage (LP) C2C12 cells that have largely lost their capacity to differentiate into myotubes. (**A**,**B**) qRT-PCR analysis results are presented as means *±* standard deviation; *n* = 3. (**A**) Exogenous *FURIN* expression in LP cells after transient transfection, normalized to endogenous FURIN levels, which were set to 1. (**B**) qRT-PCR of *HES1* levels normalized to *GAPDH* in LP myoblasts and LP myoblasts 48 h after transfection with a Furin-expressing plasmid (LP + Furin). (**C**,**D**) Representative DIC and fluorescence images of EP, LP and LP cells expressing exogenous Furin, stained with rhodamine phalloidin for F-actin (red) (**C**) and DAPI (blue) to depict nuclei and anti-myosin IIb (green) (**D**) and DAPI (blue) at 3 and/or 5 days after induction of myotube formation. In contrast to EP cells, which formed multinucleated myotubes by day 3 after induction of differentiation, LP cells remained rounded and did not differentiate. In contrast, expression of exogenous Furin restored morphological features consistent with the beginning of differentiation, including elongation and alignment of cells, and fusion into multinucleated myotubes by day 5 after induction. Scale bar = 100 µm. (**E**) Calculation of fusion index (number of myonuclei/number of total nuclei x 100%). Results are presented as means *±* standard deviation; *n* = 3. At least 700 nuclei per experiment were counted. *p*-values are indicated.

Analysis of the expression of the age-related genes in early-passage (EP) and late-passage (LP) C2C12 cells (Figure 2) showed a statistically significant negative association of *FURIN* mRNA with age, which was also confirmed at the protein level (Figure 3A,B). In addition, *NOTCH2* and *HES1* expression showed a statistically significant negative association with age. A similar trend was observed with *NOTCH3* that did not quite reach statistical significance (Figure 3C). In contrast, *GZMB* expression was positively associated with aging in the C2C12 mouse model, confirming the observations from the in silico analysis shown in Figure 2.

### 3.3. Increase in FURIN Expression Restores the Ability to Form Myotubes in Aged C2C12 Myoblasts

To determine if loss of Furin is associated with the loss of differentiation potential, LP myoblasts were transiently transfected with a Furin expression vector for 2 days before differentiation into myotubes was initiated. As shown in Figure 3A, exogenous *FURIN* mRNA expression was about 5.5- fold higher than endogenous *FURIN* mRNA at the time of differentiation initiation. The level gradually dropped after induction of differentiation but remained at about 2-fold higher than endogenous expression for the duration of the differentiation experiments (Figure 3A). To determine if the exogenous Furin was functional, we analyzed the effect of increased Furin expression on the expression level of *HES1* in LP myoblasts (Figure 3B). As mentioned above, Furin is a key protease that activates the Notch signaling pathway by cleaving the full-length NOTCH receptor that then initiates expression of the Notch target gene *HES1*. As shown in Figure 3B, as expected, *HES1* expression levels in LP myoblasts transfected with Furin showed a statistically significant increase in expression, indicating the functionality of the exogenous Furin as an activator of the NOTCH pathway [32,33].

As reported previously, early-passage C2C12 myotubes readily differentiate and fuse to form functional, multinucleated myotubes, an ability that is lost in late-passage cells after serial passaging [25,30]. However, as shown in Figure 3C,D, transient transfection of late-passage C2C12 myoblasts with a Furin expression vector restored, though with some delay, the capacity of LP myoblasts to differentiate and form myotubes. Staining for F-actin in LP cells transfected with Furin demonstrated morphological changes consistent with differentiation, i.e., elongation and alignment of cells (Figure 3C), while staining with antibodies that recognize myosin IIb heavy chain (MYH4) was used to clearly identify fused and multinucleated myotubes (Figure 3D) and to quantify the fusion index (Figure 3E) [34].

### 3.4. Increased FURIN Expression Restores Decreased MyHC_emb and IGF1 but Not MyHC-IIa Expression During Differentiation in LP Myoblasts

**Figure 4:**
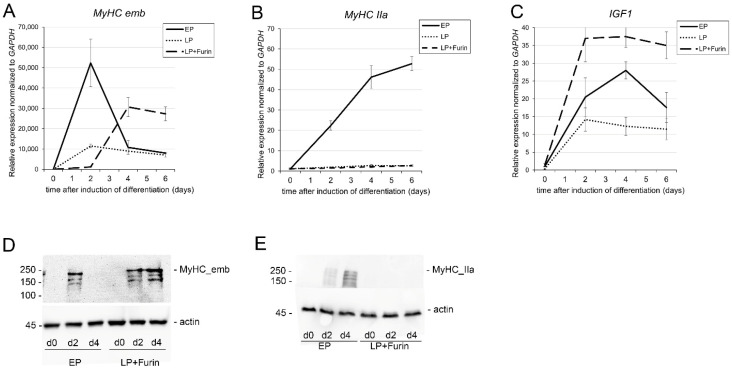
Furin expression restores the expression of embryonic MyHC (*MYH3*) and IGF1 but not adult muscle MyHC-IIa (**A**–**C**). Quantitative real-time PCR analysis of mRNA expression levels of the indicated genes normalized to *GAPDH* in EP, LP and LP cells transiently transfected with a Furin-expressing plasmid (LP + Furin) as a function of time after induction of myoblast differentiation. Results are presented as means *±* standard deviation; *n* = 3 (**D**,**E**). Representative immunoblot of total cell extract from C2C12 LP and LP cells transiently transfected with a Furin-expressing plasmid (LP + Furin) at the indicated times after induction of differentiation.

During myogenesis, muscle-specific genes, such as the genes that encode contractile proteins, including myosin heavy chains (MyHCs), are expressed. Adult mammalian skeletal muscles express four major types of myosin heavy chains (MyHCs) that are encoded by different genes: type I, IIa, IIx, and IIb [35,36]. In addition, two further isoforms, MyHC embryonic (MyHC_emb) and MyHC_neonatal (MyHC_neo), are expressed during development and are transiently expressed during skeletal muscle regeneration after injury [37,38]. Similar to human skeletal muscle, C2C12 cells start expressing MyHCs upon initiation of differentiation, including transient expression of the two developmental isoforms MyHC_emb and MyHC_neo [39]. As shown in Figure 4A,B, expression of MyHC-IIa, as well as MyHC_emb, was greatly reduced in LP cells. Interestingly, while transient expression of MyHC_emb could be rescued by Furin expression in LP cells, although somewhat delayed when compared to EP cells (Figure 4A), Furin expression had no effect on the expression of MyHC-IIa (Figure 4B). As shown in Figure 4D,E, these expression changes occurred on mRNA and protein level. In addition, we analyzed the effect of Furin expression on IGF-1 expression, another factor that is fundamentally involved in myoblast differentiation [40,41]. As shown in Figure 4C, and as previously reported, IGF-1 expression is greatly diminished in myoblasts that have undergone serial passaging, while expression of Furin leads to an overexpression of IGF-1 when compared to EP cells.

## 4. Discussion

We have identified a negative association of the cellular endoprotease FURIN with aging and demonstrate that Furin can rescue age-related loss of myogenesis by restoring myogenetic differentiation of aged myoblasts. These results highlight a role of FURIN in regulating cellular features of aging and suggest that FURIN may represent a candidate target for mitigating age-associated functional decline.

In the initial bioinformatic analysis, we focused exclusively on NOTCH and. the pre-NOTCH pathway, given the well-established knowledge of the importance of NOTCH in aging, and examined the association of the genes involved in this pathway with age in lower leg and suprapubic tissue. Based on our funding of age-related downregulation of *NOTCH2/3* and *FURIN*, and the established role of FURIN in the proteolytic activation of NOTCH precursors, we hypothesized that an age-related decrease in FURIN may lead to a decline in NOTCH receptors.

GZMB was identified as an age-associated gene inversely correlated with FURIN expression in both in silico and in vitro analyses. The *GZMB* gene encodes serine protease GZMB (Granzyme B), which is mainly expressed in NK cells and cytotoxic T lymphocytes, and capable of mediating apoptosis [42]. Elevated GZMB levels have been reported to contribute to age-related dysfunctions, such as impaired pressure injury healing [43]. In this context, GZMB accumulation appears to be a marker for age-associated dysfunction, which was reversed after the induction of FURIN expression in our experiment.

Given the broad substrate specificity of FURIN, we subsequently investigated the effect of FURIN induction on both NOTCH and other well-established aging biomarkers in the aged cell model. We used the C2C12 aging model to analyze the functional relevance of the observed reduction of Furin in age. Previous studies have shown that serial passaging of C2C12 mouse myoblasts leads to loss of their differentiation ability and ability to form multinucleated myotubes. This occurs through mechanisms that mimic aging processes in human skeletal muscles, including a reduction in IGF1 expression and alterations in the IGF-I/IGF-IR/PI3K/Akt pathways, which are critical for myoblast differentiation [25,44,45,46].

We show that transient expression of Furin can rescue the lost ability to form multinucleated myotubes, which suggests that a loss of Furin is intimately linked to the loss of differentiation potential of myoblasts during aging. Interestingly, analysis of the effect of Furin on the expression of muscle-specific MyHCs genes showed differences between adult muscle MyHC IIa, MyHC IIb, and MyHC embryonic. While expression of both MyHCs is reduced in LP cells, Furin expression can rescue MyHC embryonic and MyHC IIb (Figure 3D and Figure 4A) expression, but there is no effect on the expression of MyHC IIa (Figure 4B). While MyHC IIa is stably expressed in adult skeletal muscle, MyHC embryonic is only transiently expressed in adult muscle tissue during myogenesis, which occurs in response to injury or disease and plays a critical role in the differentiation of muscle progenitor cells and myoblasts [37,47,48]. Furin also rescued expression of IGF1, which is critically involved in myogenic differentiation during development, as well as during muscle regeneration. While the mechanisms by which Furin regulates *IGF1* expression are not known, a possible mechanism is through the activation of Insulin-like Growth Factor 1 Receptor (IGF1R), a receptor tyrosine kinase that plays a key role in cell growth, proliferation, and survival. IGF1R is initially synthesized as a precursor (proIGF1R), which requires Furin-mediated cleavage to become fully active [49]. Formation of myotubes, as well as induction of MyHC embryonic and IGF1 expression, was somewhat delayed in transfected LP myotubes compared to EP myotubes. This delay might be due to the loss or reduction of other factors involved in differentiation that might form a bottleneck despite the increase in Furin expression after transfection. Taken together, the selective effect of Furin on MyHC embryonic and IGF-1, both of which are essential for myogenesis, suggests that Furin might be involved specifically in skeletal muscle plasticity and regeneration.

Our results show a clear association between FURIN levels, NOTCH pathway activity, and myogenic function. Previous studies have already established that FURIN-mediated cleavage is necessary for NOTCH receptor maturation and activation. While our findings are consistent with this established mechanism, further experiments will be needed to verify pathway dependence. In addition, sex-specific effects on muscle aging were not addressed in this study and warrant investigation in future work.

Given the ubiquity of FURIN in mammalian cells, it has the potential to serve as an intervention target for age-related diseases in an extended scope beyond skeletal muscle aging. Reduced FURIN activity in the brain has been linked to shifts in amyloid precursor protein processing toward the amyloidogenic pathway, suggesting a potential role in neurodegenerative disease contexts [15,16]. Additionally, GZMB has also been implicated in neurodegenerative disease [50,51]. However, the extent to which modulation of FURIN can influence aging or disease progression in vivo remains to be established.

While our results indicate a potential role for FURIN in modulating age-associated declines in skeletal muscle and point the way toward new regenerative capacity, further studies are needed to verify its effects in vivo. The authors believe that restoration of FURIN function may be useful in neurodegenerative disease. Overall, we suggest FURIN as a promising candidate target for further investigation into the mechanisms driving aging or age-related decline.

## 5. Conclusions

Understanding the molecular mechanisms underlying aging is essential for developing strategies to slow or reverse age-associated functional decline. Our study identifies age-associated downregulation of FURIN and NOTCH. Such dysregulation was also observed in a replicative myoblasts aging model of C2C12. Furthermore, restoration of FURIN expression rescues myogenic function and is associated with increased NOTCH pathway activity and expression of associated biomarkers, which are amply expressed in younger cells. FURIN restoration therapy is a potential new treatment for age-related sarcopenia.

## Figures and Tables

**Table 1:** Primer sequence for qRT-PCR analysis.

Gene Name	Forward	Reverse
NOTCH2_mm	ACT TTG AGT GCC AGA GGA ATA G	CAC AGT GGT TGT CTT TGA AGT G
NOTCH3_mm	GCC ATG CAG ATG CAA GTA AAC	CGC TGT GAG ACT GAT GTC AA
FURIN_mm	GGGCATTGTAAGCTACACCTAC	CCTCGGTACACACAGATGAATG
GAPDH_mm	GTT GAA GTC GCA GGA GAC AA	TGA CAT CAA GAA GGT GGT GAA G
MyHC IIa_mm	AGGCGGCTGAGGAGCACGTA	GCGGCACAAGCAGCGTTGG
MyHC emb_mm	TCCGACAACGCCTACCAGTT	CCCGGATTCTCCGGTGAT
IGF1_mm	ATCAGCAGCCTTCCAACTC	AAGGTGAGCAAGCAGAGC
HES1_mm	TCT CCT TGG TCC TGG AAT AGT	ACT ACT GAG CAG TTG AAG GTT TAT

## Data Availability

The GTEx V8 data analyzed in Figure 1 was acquired through GTEx Portal (https://gtexportal.org/). Raw datasets used can be accessed via the GTEx Portal or our UBbox cloud storage upon request. The code has been deposited at GitHub https://github.com/UB-BiomedicalInformatics/Aging_Furin (accessed on 19 March 2026).

## Supplementary Materials

The following supporting information can be downloaded at: https://www.mdpi.com/article/10.3390/life16040588/s1, Table S1: Molecules investigated in bioinformatics analysis.
