# Supplementary material for: FURIN Stimulates NOTCH2 and NOTCH3 Pathways, Leading to Return of Function in Aged Cells
Source: Life (Basel). 2026 Apr 1;16(4):588. doi: 10.3390/life16040588 (PMC13117419; doi:10.3390/life16040588)
Supplement: Supplementary file 1 [file life-16-00588-s001.zip › life-4116594-supplementary.pdf]

ENSEMBL:ENSG00000135547 HEY2
ENSEMBL:ENSG00000164683 HEY1
ENSEMBL:ENSG00000163909 HEYL
ENSEMBL:ENSG00000136997 MYC
ENSEMBL:ENSG00000197921 HES5
ENSEMBL:ENSG00000114315 HES1
ENSEMBL:ENSG00000037280 FLT4
ENSEMBL:ENSG00000107796 ACTA2
ENSEMBL:ENSG00000206312 NOTCH4
ENSEMBL:ENST00000383264 NOTCH4
ENSEMBL:ENSG00000074181 NOTCH3
ENSEMBL:ENSG00000148400 NOTCH1
ENSEMBL:ENST00000277541 NOTCH1
ENSEMBL:ENST00000263388 NOTCH3
ENSEMBL:ENST00000256646 NOTCH2
ENSEMBL:ENSG00000207865 MIR34A
ENSEMBL:ENSG00000207811 MIR34B
ENSEMBL:ENSG00000207562 MIR34C
ENSEMBL:ENSG00000134250 NOTCH2
ENSEMBL:ENSG00000113645 WWC1
ENSEMBL:ENSG00000004399 PLXND1
ENSEMBL:ENSG00000126787 DLGAP5
ENSEMBL:ENSG00000164434 FABP7
ENSEMBL:ENSG00000171611 PTCRA
ENSEMBL:ENSG00000185630 PBX1
ENSEMBL:ENSG00000104921 FCER2
ENSEMBL:ENSG00000100453 GZMB
UniProt:O00587 MFNG
UniProt:Q9Y644 RFNG
UniProt:Q8NES3 LFNG
UniProt:Q9UM47 NOTCH3
UniProt:Q99466 NOTCH4
UniProt:Q04721 NOTCH2
UniProt:P46531 NOTCH1
UniProt:P15291 B4GALT1
UniProt:P09958 FURIN
UniProt:Q93084 ATP2A3
UniProt:O14983 ATP2A1
UniProt:P16615 ATP2A2
UniProt:Q9UBV2 SEL1L
UniProt:P20340 RAB6A
UniProt:Q11206 ST3GAL4

UniProt:Q9Y274 ST3GAL6
UniProt:Q11203 ST3GAL3
UniProt:Q15363 TMED2
UniProt:Q01196 RUNX1
UniProt:P78545 ELF3
UniProt:P24385 CCND1
UniProt:Q92793 CREBBP
UniProt:Q01094 E2F1
UniProt:O00716 E2F3
UniProt:Q14188 TFDP2
UniProt:Q14186 TFDP1
UniProt:Q06330 RBPJ
UniProt:Q13573 SNW1
UniProt:Q13495 MAMLD1
UniProt:Q8IZL2 MAML2
UniProt:Q92585 MAML1
UniProt:Q96JK9 MAML3
UniProt:Q09472 EP300
UniProt:Q92830 KAT2A
UniProt:Q92831 KAT2B
UniProt:Q9UL18 AGO1
UniProt:Q9HCK5 AGO4
UniProt:Q9H9G7 AGO3
UniProt:Q8NDV7 TNRC6A
UniProt:Q9UPQ9 TNRC6B
UniProt:Q9HCJ0 TNRC6C
UniProt:Q9HCE1 MOV10
UniProt:Q9UKV8 AGO2
UniProt:Q71DI3 H3C15
UniProt:P68431 HIST1H3G
UniProt:P84243 H3-3A
UniProt:Q99879 H2BC14
UniProt:Q96A08 H2BC1
UniProt:P58876 H2BC5
UniProt:Q93079 H2BC9
UniProt:Q16778 H2BC21
UniProt:P23527 H2BC17
UniProt:P57053 H2BC12L
UniProt:Q99877 H2BC15
UniProt:P62807 H2BC4
UniProt:P06899 H2BC11
UniProt:P33778 H2BC3
UniProt:O60814 H2BC12
UniProt:Q99880 H2BC13

UniProt:Q8N257 H2BC26
UniProt:P0C5Y9 H2AB1
UniProt:Q71UI9 H2AZ2
UniProt:P20671 H2AC7
UniProt:P16104 H2AFX
UniProt:Q9BTM1 H2AJ
UniProt:Q16777 H2AC20
UniProt:Q93077 H2AC6
UniProt:P04908 H2AC4
UniProt:Q6FI13 H2AC18
UniProt:Q99878 H2AC14
UniProt:P62805 H4C1
UniProt:Q8N6T7 SIRT6
UniProt:P04637 TP53
UniProt:P41743 PRKCI
UniProt:P0DPK3 NOTCH2NLB
UniProt:Q7Z3S9 NOTCH2NLA
UniProt:P08246 ELANE
UniProt:P0DPK4 NOTCH2NLC
UniProt:P05412 JUN
UniProt:Q9H488 POFUT1
UniProt:Q8NBL1 POGLUT1

**Table S1**. Molecules investigated in bioinformatics analysis.
